# The effect of annealing on a 3D SnO_2_/graphene foam as an advanced lithium-ion battery anode

**DOI:** 10.1038/srep19195

**Published:** 2016-01-12

**Authors:** Ran Tian, Yangyang Zhang, Zhihang Chen, Huanan Duan, Biyi Xu, Yiping Guo, Hongmei Kang, Hua Li, Hezhou Liu

**Affiliations:** 1 State Key Laboratory of Metal Matrix Composites, School of Materials Science and Engineering, Shanghai Jiao Tong University, Shanghai 200240, P.R. China

## Abstract

3D annealed SnO_2_/graphene sheet foams (ASGFs) are synthesized by *in situ* self-assembly of graphene sheets prepared by mild chemical reduction. L-ascorbyl acid is used to effectively reduce the SnO_2_ nanoparticles/graphene oxide colloidal solution and form the 3D conductive graphene networks. The annealing treatment contributes to the formation of the Sn-O-C bonds between the SnO_2_ nanoparticles and the reduced graphene sheets, which improves the electrochemical performance of the foams. The ASGF has features of typical aerogels: low density (about 19 mg cm^−3^), smooth surface and porous structure. The ASGF anodes exhibit good specific capacity, excellent cycling stability and superior rate capability. The first reversible specific capacity is as high as 984.2 mAh g^−1^ at a specific current of 200 mA g^−1^. Even at the high specific current of 1000 mA g^−1^ after 150 cycles, the reversible specific capacity of ASGF is still as high as 533.7 mAh g^−1^, about twice as much as that of SGF (297.6 mAh g^−1^) after the same test. This synthesis method can be scaled up to prepare other metal oxides particles/ graphene sheet foams for high performance lithium-ion batteries, supercapacitors, and catalysts, etc.

The continuous surge of the energy demand emerging large-scale energy applications such as low-emission electric vehicles, renewable power plants, and electric grids boost a great deal of interest in seeking high-performance and long-life maintaining power devices. Lithium-ion batteries (LIBs), as one of the most useful power devices, have been widely used around the world^1^,^2^. Nowadays, the graphite anode has many problems such as low capacity density and bad cycling performance. It is one of the reasons that hinder the development of the lithium-ion batteries, so the research of the next generation of the lithium-ion battery anodes to replace graphite is the key point to develop the lithium-ion batteries^3^.

Various electrochemically active materials such as SnO_2_, Fe_2_O_3_, Fe_3_O_4_, Si, Ge and Sn in the form of nanoparticles have been studied to this end^4^,^5^. However, the practical use of these materials is severely hampered by huge volume change during discharge/charge process, which causes pulverization, unstable solid electrolyte interface (SEI), particle aggregation, and subsequently rapid capacity deterioration^4^. Graphene, a single-atom-thick sheet of honeycomb carbon lattice with high electrical conductivity and large specific surface area (theoretical value 2620 m^2^ g^−1^), can form nano-composite materials with the above-mentioned nanoparticles to alleviate the adverse mechanical effects and improve the electrochemical performance^6^,^7^. In the previous work, the research mostly focuses on the 2D graphene sheet (GS)/nano-metallic oxide composite materials, such as SnO_2_/GS^8^,^9^,^10^,^11^,^12^,^13^,^14^, Fe_2_O_3_/GS^15^,^16^,^17^, NiO/GS^18^, Co_3_O_4_/GS^19^, and ZnO/GS^20^. Therein the GS did prevent the aggregation of nanoparticles to a certain extent, but the contact between active materials and the electrolyte need to be improved to address the issue of limited electron and ion transport.

Recently, 3D GS aerogels foam has attracted much attention due to their low density, high surface areas, 3D conductive networks, and microporous and mesoporous structures^21^. The 3D GS foam (GSF) can be prepared by chemical reduction (using reducing agents including HI, NaHSO_3_, L-ascorbyl acid and so on^21^,^22^,^23^), hydrothermal reduction^24^,^25^ and chemical vapor deposition (CVD)^26^. A variety of the nanoparticles/GS foams such as SnO_2_/GSF^27^,^28^,^29^, Co_3_O_4_/GSF^30^, Fe_2_O_3_/GSF^31^,^32^ have been successfully synthesized. For example, Chen at al. prepared the 3D composite aerogels made of GS and Fe_3_O_4_ nanoparticles using NaHSO_3_ as a reducing agent and found that the materials have excellent electrochemical performance^33^. In this method, the GSs self-assemble into a 3D macroscopical structure by the π−π stacking interactions; metal ions are adsorbed on the surface of GS under the electrostatic attraction of the reduction of graphene oxide (GO) and *in situ* deposition to form metal-oxide nanoparticles^33^. In spite of the success, the use of pressure containers and high temperature prevents this method from becoming a commercial process. On contrast, the chemical reduction self-assembly method can react at a temperature under the water boiling point to form GSF; hence the experiment can be carried out without explosion hazard using containers such as glass bottles and common stainless steel pot. Moreover, the l-ascorbyl acid can reduce GO in 8 hours that is much shorter than 12 hours used in the hydrothermal method^34^. Although the chemical reduction self-assembly is easy to realize, it has shortcomings such as the chemical separation between particles and GS, wiping off the reducing agent and so on. Further, the particles attract to the GS by hydrogen bonds and Van der Waals force that usually lead to inferior ionic and electronic transport. Thus, how to enhance the connection of the particles and GS is one of the key points to improve the electrochemical performance, but little research has been done in this area.

In this study, we adopted the chemical self-assembly route to synthesize the SnO_2_ nanoparticles/GS foam and found that annealing process can significantly enhance the interaction between the SnO_2_ nanoparticles and the GS. The composite materials were used as anode materials for lithium ion batteries and exhibited excellent cycling stability (844.8 mAh g^−1^ after 50 cycles at 200 mA g^−1^ and 533.7 mAh g^−1^ after 150 cycles at 1000 mA g^−1^). After the annealing treatment, the electrochemical performance of the materials has been greatly improved especially at high specific currents (91.2% increased at 3000 mA g^−1^) because the formation of Sn-O-C bonds leads to the synergistic effect between the SnO_2_ nanoparticles and GS.

## Results

**Figure 1:**
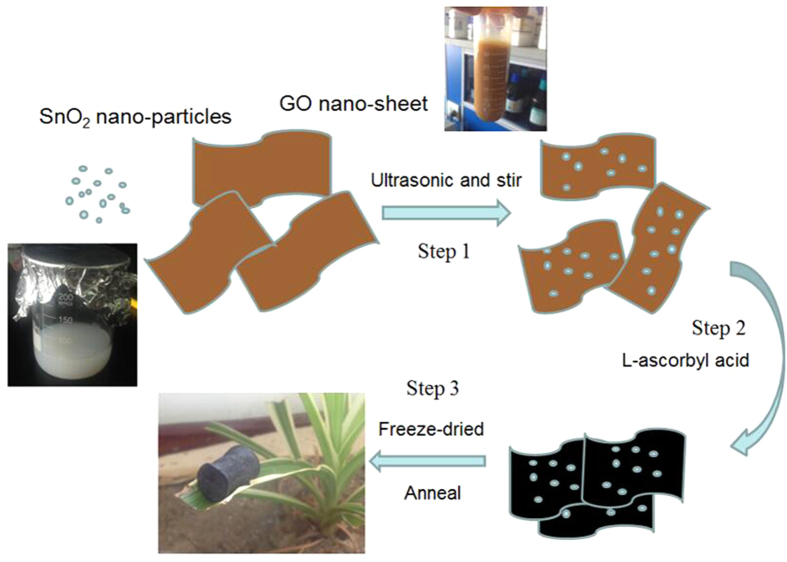
Schematic Illustration of Preparation of ASGF.

The SnO_2_/GS Foam was fabricated by a chemical self-assembly strategy and a subsequent freeze-drying process (Fig. 1). At step 1, from the collochemistry, the metal oxide such as Fe_2_O_3_, TiO_2_ and SnO_2_ colloid was positively charged; the GO was negatively charged because GO has many oxygen containing functional groups on the surface^35^. The GO and SnO_2_ nanoparticles were attracted by the electrostatic force so that the SnO_2_ nanoparticles can distribute on the surface of GO and not departure by the ultrasonication and stir. At step 2, L-ascorbyl acid was used to reduce oxygen containing functional groups (e.g. carboxyl) on the surface GO, producing *in situ* reduced GO (rGO). rGO has smaller solubility in water than GO because the reduction of polar oxygen containing functional groups makes GO less hydrophilic. So, as GO sheet began to turn into the rGO, the delocalized π-bond’s conjugative effect would be increased and enlarged. The freshly formed rGO sheets would stack on other rGO sheets as a result of the π–π stacking interactions and self-assembled into a 3D structure. After the chemical reaction and at step 3, there are still many oxygen containing functional groups left on rGO sheets, thus, the SnO_2_ nanoparticles with polar surfaces would interact with those functional groups via hydrogen bonding. By annealing at 550 °C, the hydrogen bonds may turn into oxygen bridges between SnO_2_ and rGO, forming Sn-O-C bonds. Therefore, the SnO_2_ nanoparticles are anchored strongly on the graphene surface through a C-O-Sn bridge, which facilitates the electron transfer and improve the electrode stability. Finally, we obtain a porous ASGF with relative density of ~19 mg cm^−3^.

**Figure 2:**
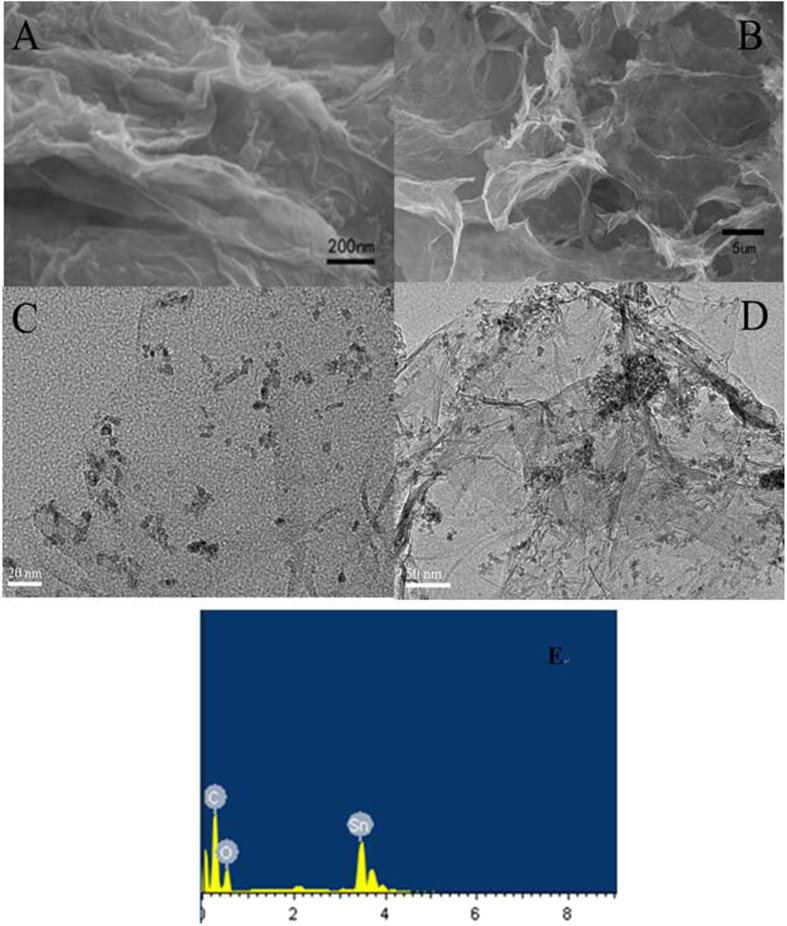
Morphology characterization of ASGF. SEM images (**A,B**) and EDX spectrum (**E**); TEM images with different magnifications (**C,D**)

The morphologies of the as-prepared ASGF were investigated by SEM and TEM. Typical SEM images in Fig. 2A,B show that ASGF possess a 3D structure with interconnected pores ranging from several nanometers to several micrometers. Moreover, the energy dispersive X-ray spectroscopy (EDX) measurement of the ASGF reveals that presence of Sn, O, and C. (Fig. 2E). The TEM images (Fig. 2C,D) show that the SnO_2_ nanoparticles with size in the range of 6–12 nm are distributed uniformly on the surface of a continuous 3D porous network made of ultrathin graphene sheets, in good agreement with the SEM observation above.

**Figure 3:**
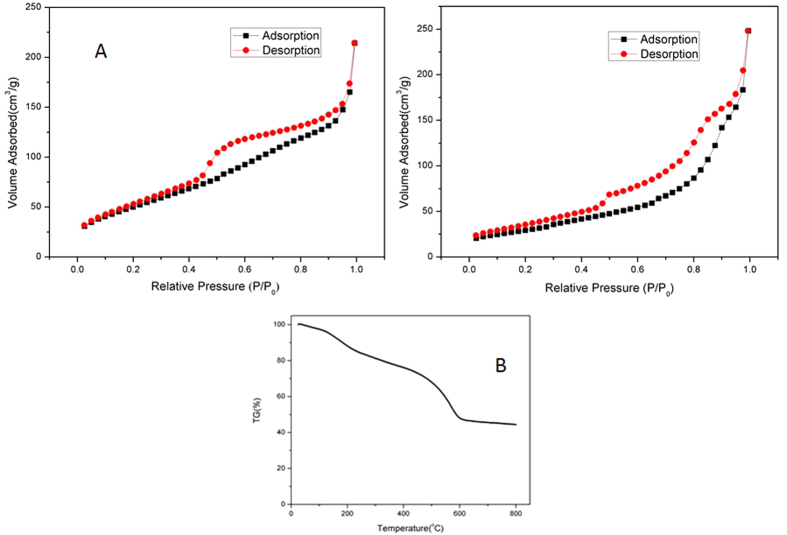
Nitrogen adsorption and desorption isotherms of ASGF (**A**) and SGF (**B**); TGA profile of SGF (**C**).

The porous nature of SnO_2_-graphene architecture is further validated by nitrogen physisorption measurements and the results are shown in Fig. 3a. Clearly, for both the SGF and ASGF, the N_2_ adsorption-desorption isotherms exhibit a typical II hysteresis loop at a relative pressure between 0.42 and 0.95, characteristic to pores with different pore sizes^36^. The surface area of the SGF and ASGF was determined to be 188 and 109 m^2^ g^−1^ by Brunauer–Emmett–Teller (BET) calculations, respectively. To gain insight into the chemical composition, thermo-gravimetric analysis (TGA) was performed on the 3D SnO_2_-graphene foam and typical result of SGF is shown in Fig. 3B. The sample is annealed under air at the 10 °C min^−1^ heating rate to remove the moisture and oxidize carbon to CO_2_. From the TGA data, the original content of SnO_2_ is calculated to be 45.56 wt%. In combination with the analysis based on SEM and TEM images, we can safely conclude that the SnO_2_-garphene foam have a 3D graphene architectures that give rise to high surface areas and multilevel porous structures. It is this unique morphology that can greatly facilitate the access of electrolyte and the fast diffusion of lithium ion and electrons during lithium storage.

**Figure 4:**
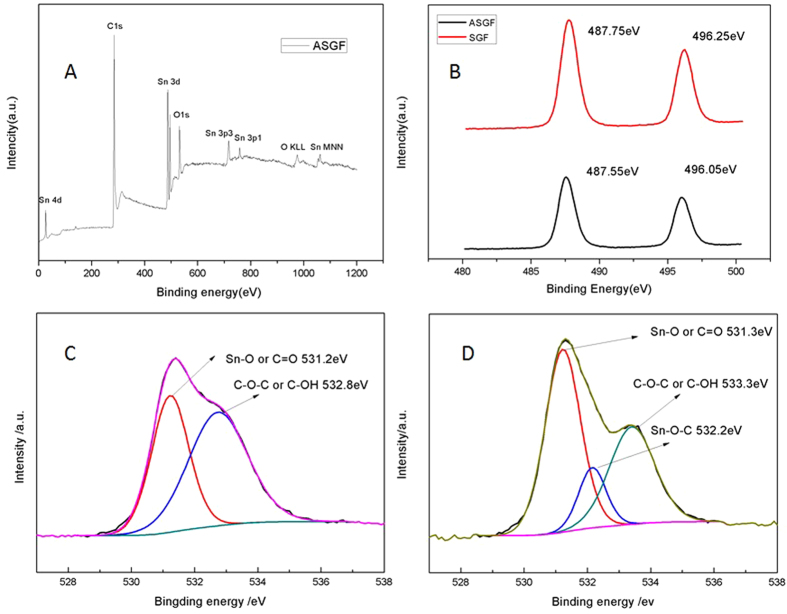
XPS spectra of SGF and ASGF. (**A**) general XPS spectrum of ASGF; (**B**) Sn 3d XPS spectrum; (**C**) O1s XPS spectrum of SGF; (**D**) O1 s XPS spectrum of ASGF

**Figure 5:**
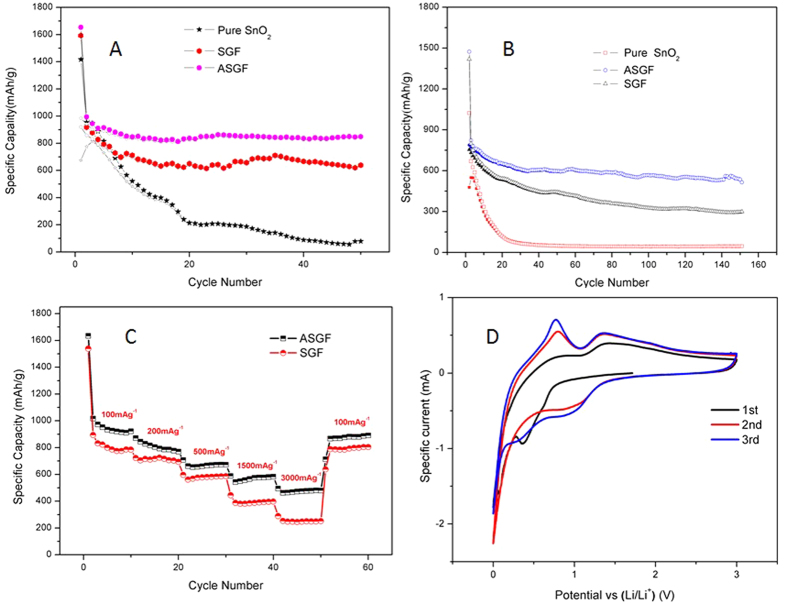
Cycling performances of ASGF, SGF, and pure SnO_2_ nanoparticles at specific currents of 200 mA g^−1^ (**A**) and 1000 mA g^−1^ (**B**); rate capability of ASGF and SGF at current densities from 100 mA g^−1^ to 3000 mA g^−1^ (**C**); CV curves of the first three cycles of ASGF at a scanning rate of 0.5 mV s^−1^ (**D**).

The surface chemistry of the ASGF was characterized using XPS and the results are depicted in Fig. 4. As shown in Fig. 4A, the general XPS spectrum proves the presence of carbon, oxygen and tin, and no other elements are detected. The peaks of Sn 3d, 4d, 3p and 4s from SnO_2_ are observed. The peak of C 1s is attributed mainly to graphene. The Sn 3d spectra of both SGF and ASGF, as shown in Fig. 4B, consist of two peaks at around 487.6 eV and 496.l eV, corresponding to Sn 3d_5/2_ and Sn 3d_3/2_ spin-orbit peaks of SnO_2_, respectively, confirming the formation of SnO_2_ nanoparticles on the surface of graphene sheets, the minute difference between SGF and ASGF indicates the different chemical environment of SnO_2_ nanoparticles^37^. Figure 4C shows that the O 1s core level peak of SGF can be resolved into two components centered at 531.2 eV, and 532.8 eV, which can be assigned to Sn-O and/or C=O bonds and C-OH and/or C-O-C groups (hydroxyl and/or epoxy), respectively. On contrast, Fig. 4D shows that the O 1s core level peak of ASGF consists of three components centered at 531.3, 532.2 and 533.3 eV, which can be assigned to Sn-O and/or C=O bonds, Sn-C-O bonds, and C-OH and/or C-O-C groups (hydroxyl and/or epoxy)^38^. Clearly, the annealing treatment induces the formation of new bonds — the Sn-C-O bonds, confirming the strong interaction between the SnO_2_ nanoparticles and the graphene surface, which is the key to have the synergistic effect to improve the electrochemical properties^38^,^39^. Besides, the annealing treatment also decrease the C-OH and/or C-O-C groups (hydroxyl and/or epoxy) in the rGO that also helps enhance the electrical conductivity of ASGF^40^. The electrochemical properties of ASGF and SGF were systematically evaluated by galvanostatic discharge (lithium insertion)-charge (lithium extraction) measurements. Figure 5A,B compare the cycling performance for ASGF, SGF, and pure SnO_2_ nanoparticles at specific currents of 200 mA g^−1^ and 1000 mAh g^−1^ between 0.01 and 3 V vs. Li^+^/Li. The initial discharge and charge capacities of the ASGF at 200 mA g^−1^ are 1653 mAh g^−1^ and 984.2 mAh g^−1^, respectively, with a Coulombic efficiency (CE) around 60%. The CE of the second cycle increases to be 94.7% and maintains thereafter about 97% after 3 cycles (Fig. S1). After 50 cycles at 200 mA g^−1^, the ASGF electrode still exhibits a reversible capacity of 845 mAh g^−1^, which is 89.7% of the value of the second cycle.

By contrast in Fig. 5A, the cycling profiles for the SGF and the pure SnO_2_ nanoparticles show continuous and progressive capacity decay along with cycling processes. In specific, the discharge and charge capacities of the SGF after the first cycle are 1592.7 mAh g^−1^ and 919.8 mAh g^−1^, respectively; after the second cycle, are 918.1 and 858.4 mAh g^−1^, respectively; after 50 cycles, 638.3 mAh g^−1^ and 634.6 mAh g^−1^, respectively. The capacity retention of the SGF after the 50^th^ cycle is 73.9% with respect to that after the second cycle. For the pure SnO_2_ nanoparticles, the capacity retention is much worse compared to SGF, and the specific capacity drops to as low as 201.8 mAh g^−1^ after 25 cycles and remains thereafter.

Figure 5B shows that the capacity-retention advantage of the ASGF over the SGF and the pure SnO_2_ nanoparticles is more obvious at higher current densities. After 150 cycles at the specific current of 1000 mAh g^−1^, the capacity of ASGF electrode is 533.7 mAh g^−1^ which is much better than SGF (294.8 mAh g^−1^) and pure SnO_2_ nanoparticles (44.1 mAh g^−1^).

Figure 5C shows the rate capability of the ASGF and SGF electrodes. As the current densities increase stepwise from 100 to 200, 500, 1500 and 3000 mA g^−1^, the ASGF electrode delivers stable capacities varying from 922.0 to 770.5, 672.3, 582.8 and 480.3 mAh g^−1^, respectively; the SGF electrode from 784.5 to 693.7, 587.0, 396.9 and 251.2 mAh g^−1^, respectively. The specific capacities of the ASGF electrode is 17.4%, 11.1%, 14.5%, 46.8%, 91.2% higher than those of the SGF electrode at 100, 200, 500, 1500 and 3000 mA g^−1^. When the specific current returns to 100 mA g^−1^, the capacity recovers to 890.9 mA g^−1^ for the ASGF electrode, close to that after the 10th cycle at 100 mA g^−1^. The result shows that the ASGF has higher reversible capacity at the high current rates and better rate capability compared with the SGF.

Figure S2 shows the charge-discharge profiles of the ASGF(A) and SGF(B) electrode at a specific current of 200 mA g^−1^. The voltage profiles present sloping lines during both charge and discharge processes, in accordance with the broad peaks observed during CV scans. Moreover, both charge and discharge profiles exhibit little change from the second to the 50^th^ cycles, demonstrating that the ASGF electrodes are very stable during cycling^41^,^42^.

The Fig. 5D presents the CV curves of the first three cycles of the ASGF from 0 to 3 V vs. Li^+^/Li at a scanning rate of 0.5 mV s^−1^. For the first cycle, an obvious reduction peak is present near 0.4V, which is considered to be the formation of SEI layers on the surface of the SnO_2_ and the GS (Eq. 1). It is generally accepted that this reaction is not reversible and should be responsible for the large irreversible capacity loss during the first cycle^43^,^44^. After the first cycle, the curves are more consistent. For the third cycle in particular, two broad reduction peaks around the 1 and 0.3 V can be ascribed to the reduction of the SnO_2_ (Eq. 2 and Eq. 3) and the alloying reaction between Sn and Li and the lithium-ion insertion on the GS surface (Eq. 4 and Eq. 5)^43^. The oxidation peaks around 0.8 V and 1.4 V may correspond to the dealloying process of Li_x_Sn alloy and the transition of Li_2_O to SnO_x_^38^. It shall be noted that Eq. 2 and 3 are usually considered to be irreversible, but the SnO_2_ nanoparticles have the size effect to decrease the activation energy of this electrochemistry reaction and enable its reversibility; similar effects have been observed in other transitional metal oxide nanoparticles such as Fe_3_O_4_, NiO, CuO^45^,^46^,^47^.


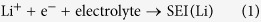



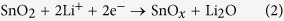



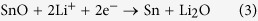



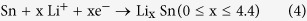



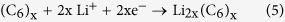


## Discussion

Comparing with the SnO_2_/GS composites^11^, the ASGF has higher reversible capacity at high current rates because of the 3D GS conductive frameworks with microporous and mesoporous structures that facilitate the ion and electron transport during the electrochemical reactions^27^. To synthesize such 3D porous nanostructured electrodes, a commonly used method is hydrothermal reduction. If this method is applied to produce graphene sheet foams in large scale, large size Teflon-lined autoclaves are required considering the limited GO’s concentration in water, which brings up the cost. Besides, the high temperature and pressure in the hydrothermal reduction process can cause safety concerns^27^,^28^. On contrast, the present synthesis route – combination of mild chemical reduction and heat treatment – uses common glass bottles or stainless steel pot, uses a much lower synthesis temperature, and is safe, which is advantageous for mass production.

During the cycling test of the ASGF (Fig. 5A,B), the large capacity loss (~40%) of the first cycle is generally attributed to the irreversible formation of the SEI layer on the surface of the nanohybrids during the first discharge process^47^,^48^,^49^. Moreover, the initial discharge capacities (1653 mAh g^−1^) is higher than the theoretical capacity of SnO_2_ (1494 mAh g^−1^) and graphene sheet (744 mAh g^−1^), which can be attributed to the oxygen containing functional groups that can react with lithium ions, exhibit certain capacity, and enhance the irreversible capacity^50^. We note that there was an increase of the capacity of the ASGF after the 38^th^ cycle in Fig. 5A. The reasons may have two folds: 1) the delayed infiltration of the electrolyte into the nanohybrids^9^,^27^,^45^,^51^; 2) the catalytic capability of the SnO_2_ and Sn nanoparticles produced during the cycling process to enable reversible conversions of the SEI layer^52^.

**Figure 6:**
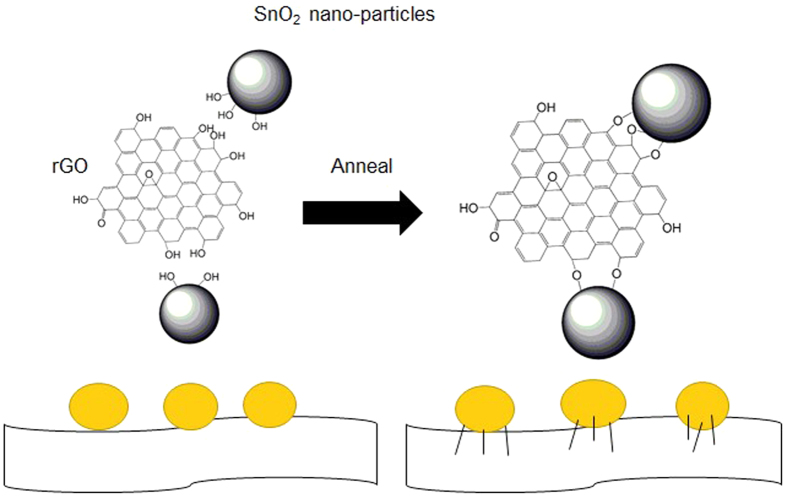
Schematic illustration of the annealing treatment.

The effect of annealing is schematically shown in Fig. 6. The main reason of the good specific capacities, capacity retention and stability of ASGF is the synergistic effect between the GS and SnO_2_ nanoparticles. As evidenced in the XPS data, without annealing there are no chemical bonds between the SnO_2_ nanoparticles and the GS in the SGF. Weak interactions such as hydrogen bonds and/or van der Waals force have been suggested^9^; these weak interactions may cause detachment and loss of SnO_2_ nanoparticles during Li^+^ insertion and extraction, leading to decaying capacity during cycling. After annealing, on contrast, chemical bonds (i.e. Sn-O-C bonds) form between the SnO_2_ nanoparticles and the GS; these Sn-O-C bonds set up oxygen bridges between SnO_2_ nanoparticles and GS. The oxygen bridges will have two folds of effects on the hybrid foam: facile electronic transport and strong attachment of SnO_2_ nanoparticles on the GS surface, which will lead to easy binding/difficult dissociating characteristic of Sn adatoms on the oxygenated graphene, facilitates fast electron hopping from graphene to SnO_2_, and thus promotes the reversible lithiation and delithiation of SnO_2_ at the higher specific current. A secondary reason may be that the annealing treatment improves the electrical conductivity of the GS by itself. Figure 4C and D show decreasing density of the peaks associated with the C-OH and/or C-O-C groups (hydroxyl and/or epoxy) after the annealing treatment, which suggests the removal of oxygen containing functional groups. This contributes to fast electron transfer in the GS and subsequently leads to superior rate capability and cycling stability of ASGF^40^. Indeed as shown in Fig. 5C, the specific capacities of the ASGF electrodes are much higher than those of the SGF electrodes at the high specific current; as shown in Fig. 5A,B, the ASGF electrodes have better cycling stability than the SGF ones.

In summary, we have successfully prepared the SnO_2_/GS foam by *in situ* self-assembly of graphene prepared by a mild chemical reduction, which is suitable for mass production. After annealing treatment at 550 °C, Sn-C-O bonds form between SnO_2_ nanoparticles and GS. The as-annealed foams exhibit superior rate capability and capacity retention in comparison to the samples without annealing, the excellent electrochemical performance of the annealed SnO_2_/graphene foam arises from the following three aspects: (1) the 3D GS frameworks with microporous and mesoporous structures that facilitate the ion and electron transport throughout the electrode; (2) the hybrid structure of the SnO_2_ nanoparticles on the surface of the GS that improves the tolerance to change and alleviates the agglomeration and pulverization; and (3) the formation of Sn-O-C bonds and the removal of oxygen containing functional groups during the annealing that facilitates electron transfer and improves long-term stability. This work can be scaled up to prepare various metal oxide nanoparticles/GS composites for a broad range of applications such as batteries, supercapacitors, catalysts, and so on.

## Methods

### Preparation of SnO_2_ nanoparticle suspension

The homogeneous nano-size SnO_2_ suspension was synthesized by hydrothermal method^13^. 2.0 g SnCl_4_•5H_2_O (Tianjin Kermel Chemical Reagent Co., Ltd., China) was dissolved in 80 mL deionized water. The settled solution was transferred into two 50 ml Teflon-lined stainless steel autoclaves and maintained at 160 °C for 18 h. After cooling and centrifugation, the collected white precipitate (SnO_2_ nanoparticles) was re-dispersed in 100 ml ethanol to form a homogeneous suspension named SnO_2_-ES.

### Synthesis of annealed SnO_2_/GS Foam

Graphene oxide (GO) was prepared by a modified Hummers method^53^. 15 ml SnO_2_-ES was added into 50 ml graphene oxide (GO) dispersion (about 2.5 mg ml^−1^) under stirring and kept stirring for 3 h, followed by ultrasonicating for 1 h. 250 mg L-ascorbyl acid was added into the mixed suspension. After that, the mixed suspension was moved into the oven at 75 °C for 8 h to reduce GO and induce self-assembly process to obtain a 3D SnO_2_/GS monolith in a glass bottle. Then the monolith was taken out, washed repeatedly with deionized water for 2 days, and freeze-dried into a SGF. Followed by thermal treatment at 550 °C for 2 h under argon atmosphere, the SGS was turned into annealed SGF (ASGF).

### Materials characterization

The morphology of samples was characterized by the transmission electron microscope (JEM-2100F, JEOL, Tokyo, Japan) and the field-emission scanning electron microscope (FEI-Sirion 200). Thermal gravimetic analysis (TGA) was conducted in air at a heating rate of 10 °C min^−1^. X-ray photoelectron spectroscopic (XPS) measurements were performed on a Kratos AXIS Ultra DLD spectrometer with a monochromatic Al Ka X-ray source. Nitrogen absorption and desorption measurements were performed with an Auto sorb IQ instrument. The surface areas were calculated by the Brunauer-Emmett-Teller (BET) method.

### Electrochemical measurements

The electrochemical experiments were carried out using coin-type cells. The ASGF and SGF are smashed to small chippings to form the active material. The electrodes were prepared by mixing 70 wt% active material, 20 wt% conducting carbon black, and 10 wt% polyvinylidene fluoride binder in N-methyl-2-pyrrolidone. The resultant slurry was uniformly pasted on Cu foil with a blade, dried at 100 °C in a vacuum oven. The loading mass of active materials (SnO_2_/graphene) on current collectors is about 1.0 mg cm^−2^. For comparison, pure SnO_2_ nanoparticles were used as the control group.

The electrochemical properties of the electrodes were characterized at room temperature. Li foil was used as the counter electrode. The Celgard 2325 microporous membrane was used as separator. The electrolyte was 1 M LiPF_6_ in a 50:50 w/w mixture of ethylene carbonate (EC) and dimethyl carbonate (DMC). CR2025-type coin cell assembly was carried out in a Mikrouna glovebox with the concentrations of moisture and oxygen below 1 ppm. Galvanostatic cycling test was done in a voltage range from 3.0 to 0.01 V using a LAND CT2001A battery tester. Cyclic voltammetry (CV) was carried out on an electrochemical workstation (Bio-Logic, France) in a voltage range of 0–3.0 V v.s. Li/Li^+^ with a scan rate of 0.5 mV s^−1^.

## Additional Information

**How to cite this article**: Tian, R. *et al.* The effect of annealing on a 3D SnO_2_/graphene foam as an advanced lithium-ion battery anode. *Sci. Rep.* **6**, 19195; doi: 10.1038/srep19195 (2016).

## Supplementary Material

Supporting Information
