# Supplementary material for: The effect of annealing on a 3D SnO2/graphene foam as an advanced lithium-ion battery anode
Source: Sci Rep. 2016 Jan 12;6:19195. doi: 10.1038/srep19195 (PMC4709726; doi:10.1038/srep19195)
Supplement: Supporting Information [file srep19195-s1.doc]

**Supplementary Information**

**The effect of annealing on a 3D SnO_2_/graphene foam as an advanced lithium-ion battery anode**

Ran Tian, Yangyang Zhang, Zhihang Chen, Huanan Duan*, Biyi Xu, Yiping Guo, Hongmei Kang, Hua Li, Hezhou Liu*

State Key Laboratory of Metal Matrix Composites, School of Materials Science and Engineering, Shanghai Jiao Tong University, Shanghai 200240, P.R. China

**The supporting information includes the following content**

Fig S1 The coulombic efficiency of ASGF at 200mA g^-1^

Fig S2 The galvanostatic discharge-charge profiles of different cycles for ASGF(A) and SGF(B) at 200mA g^-1^


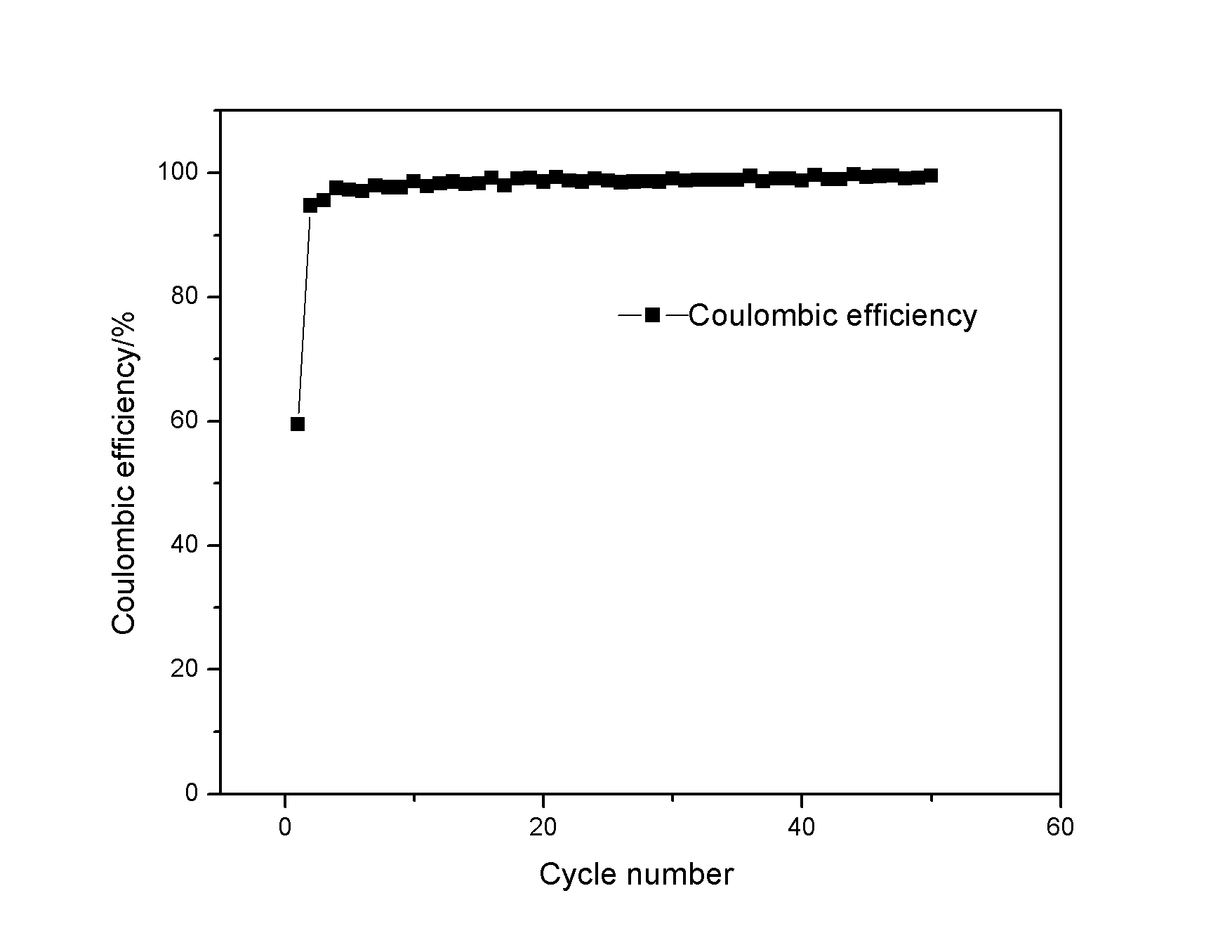


Fig S1 The coulombic efficiency of ASGF at 200mA g^-1^


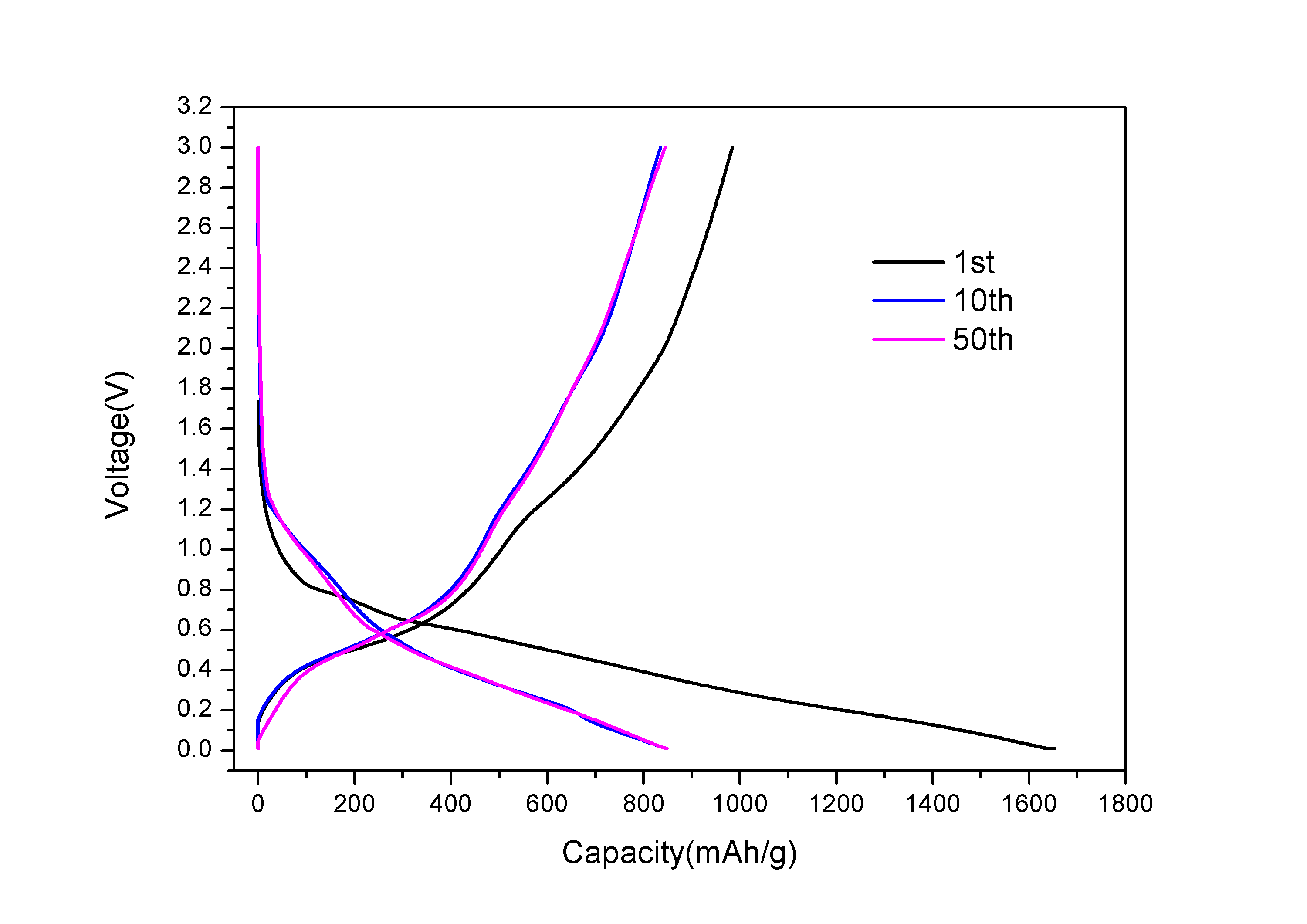


A


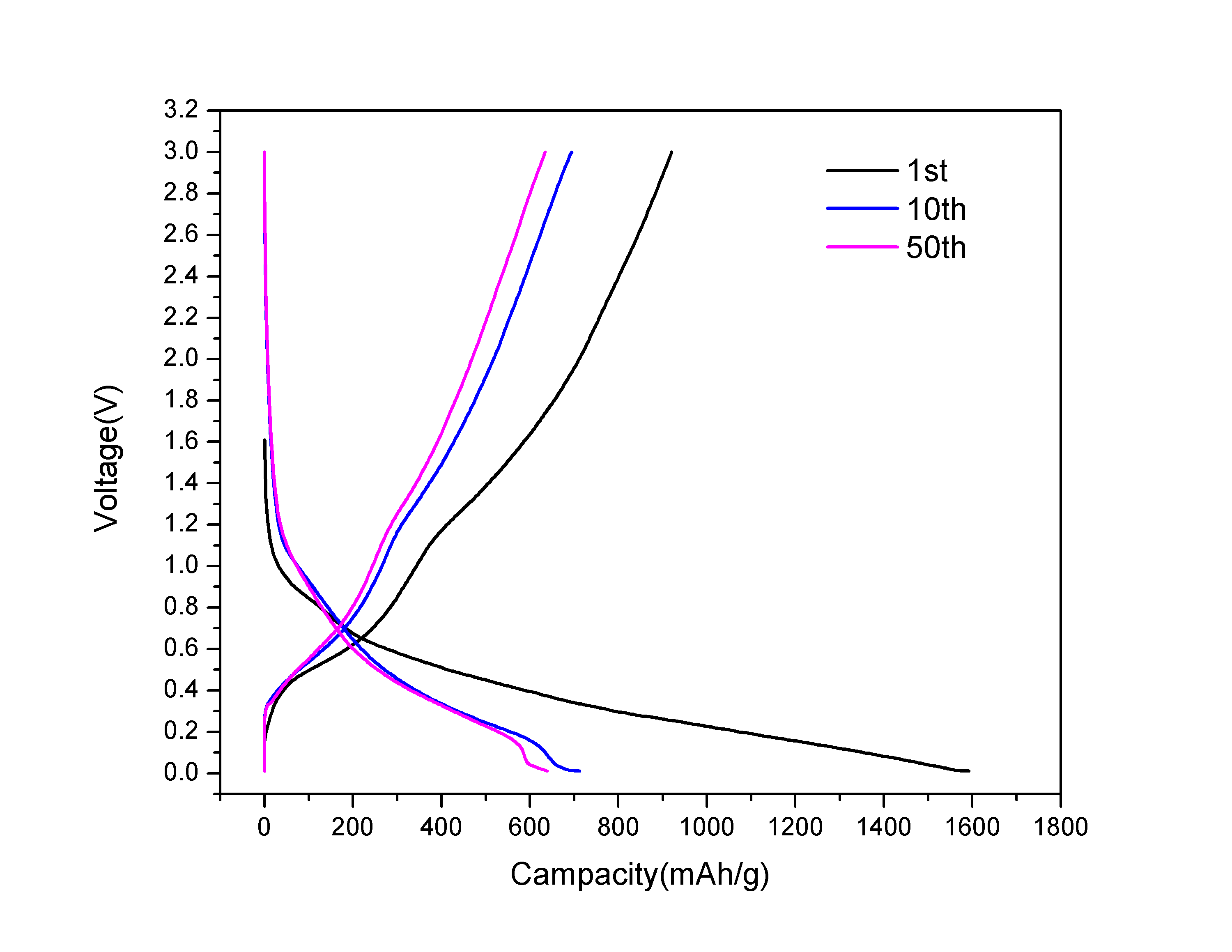


B

Fig S2 The galvanostatic discharge-charge profiles of different cycles for ASGF(A) and SGF(B) at 200mA g^-1^
